# Cranial ultrasound in preterm infants ≤ 32 weeks gestation—novel insights from the use of very high-frequency (18-5 MHz) transducers: a case series

**DOI:** 10.1007/s00431-024-05627-y

**Published:** 2024-06-03

**Authors:** Francesca Miselli, Isotta Guidotti, Marianna Di Martino, Luca Bedetti, Chiara Minotti, Eugenio Spaggiari, Giovanni Malmusi, Licia Lugli, Lucia Corso, Alberto Berardi

**Affiliations:** 1 PhD Program in Clinical and Experimental Medicine, University of Modena and Reggio Emilia, Modena, Italy; 2 Neonatal Intensive Care Unit, Women’s and Children’s Health Department, University Hospital of Modena, Via del Pozzo, 41124 Modena, Italy; 3 School of Pediatrics Residency, University of Modena and Reggio Emilia, 41224 Modena, Italy; 4 Neonatal Intensive Care Unit, Italy Department of Obstetrics and Pediatrics, Azienda USL-IRCCS di Reggio Emilia, Reggio Emilia, Italy; 5 Department of Medical and Surgical Sciences for Mother, Child and Adult, University of Modena and Reggio Emilia, Modena, Italy

**Keywords:** Cranial ultrasound, Transfontanellar, Preterm, Infant, Very high-frequency, Case series

## Abstract

The quality of cranial ultrasound has improved over time, with advancing technology leading to higher resolution, faster image processing, digital display, and back-up. However, some brain lesions may remain difficult to characterize: since higher frequencies result in greater spatial resolution, the use of additional transducers may overcome some of these limitations. The very high-frequency transducers (18-5 MHz) are currently employed for small parts and lung ultrasound. Here we report the first case series comparing the very high-frequency probes (18-5 MHz) with standard micro-convex probes (8-5 MHz) for cranial ultrasound in preterm infants. In this case series, we compared cranial ultrasound images obtained with a micro-convex transducer (8-5 MHz) and those obtained with a very high-frequency (18-5 MHz) linear array transducer in 13 preterm infants ≤ 32 weeks gestation (9 with cerebral abnormalities and 4 with normal findings). Ultrasound examinations using the very high-frequency linear transducer and the standard medium-frequency micro-convex transducer were performed simultaneously. We also compared ultrasound findings with brain MRI images obtained at term corrected age. Ultrasound images obtained with the very high-frequency (18-5 MHz) transducer showed high quality and accuracy. Notably, despite their higher frequency and expected limited penetration capacity, brain size is small enough in preterm infants, so that brain structures are close to the transducer, allowing for complete evaluation.

*    Conclusion*: We propose the routine use of very high-frequency linear probes as a complementary scanning modality for cranial ultrasound in preterm infants ≤ 32 weeks gestation.

**Table:** 

**What is Known:** • *Brain lesions in preterm infants may remain insufficiently defined through conventional cranial ultrasound scan.* • *Higher frequency probes offer better spatial resolution but have a narrower filed of exploration and limited penetration capacity.*
**What is New:** • *Very high-frequency probes were compared with standard medium-frequency probes for cranial ultrasound in infants ≤ 32 weeks' gestation.* • *Thanks to the smaller skull size of preterm infants, the new very high-frequency transducers allowed a complete and accurate evaluation.*

**Supplementary Information:**

The online version contains supplementary material available at 10.1007/s00431-024-05627-y.

## Introduction

Since its introduction in the late 1970s, cranial ultrasound (US) has been widely used in neonatal intensive care units (NICUs) to investigate congenital and acquired brain abnormalities in both full-term and preterm neonates. This radiation-free technique has been increasingly employed and is now the first-line neuroimaging modality to study the neonatal brain. In the NICU context, bedside cranial US studies can be serially performed with acceptable disturbance to the infant and do not require patient transport nor sedation [1, 2]. The procedure provides quick images in real time and can be performed directly after birth [2].

Several indications are established for the study of the neonatal brain. Cranial US allows the diagnosis of both germinal matrix (GMH) and intraventricular haemorrhage (IVH) of preterm infants, and also their follow-up (monitoring for complications, e.g., hydrocephalus or parenchymal haemorrhage). Indeed, cranial US is an excellent tool to detect ventricular enlargement and white matter injury (WMI). Its role is also crucial in all critical neonates that cannot be investigated through brain magnetic resonance imaging (MRI) because of haemodynamic or respiratory instability [1, 3].

Images of good quality can be obtained by using a curved or phased array transducer of medium-frequency (5–7.5 MHz), which penetrates the neonatal brain with adequate resolution. Scans are usually performed through the anterior fontanelle. However, for regions not easily approachable through this fontanelle, alternative acoustic windows with adapted settings are used. These alternative windows enable visualization of challenging areas and aid in obtaining a more detailed characterization of lesions already detected through the anterior fontanelle (e.g., in the context of IVH grading).

The quality of cranial US has dramatically improved over time, with advancing technology leading to higher resolution, faster image processing, digital display, and back-up [1, 3]. Despite improved resolution and depth penetration, some minor lesions remain difficult to assess: detailed assessment of superficial structures located at the convexity of the cerebral hemispheres, including the subarachnoid spaces, cortex, and subcortical white matter, is difficult with standard medium-frequency transducers [4]. Also, the state of myelination, a major prognostic index in case of preterm birth or suspected brain injury, is not adequately depicted [5]: diffuse WMI is insufficiently detected, and the normal white matter echogenicity on cranial US is not a good predictor of normal white matter signal intensity on MRI [6].

Ideally, the use of additional transducers may overcome some of these limitations, since high-frequency (> 9 MHz) transducers, preferably linear, improve imaging resolution and are now highly recommended [3, 7]. The potential role of additional scanning with frequency > 9 MHz has been so far proposed for selected cases, including (near) term neonates with (suspected) hypoxic–ischemic brain injury, suspicion of focal infarction or abnormalities in the meningeal spaces, superior sagittal sinus, or the cerebral cortex [3, 4]. However, it is considered that the main limitation of high-frequency probes is the shallow depth of view [3]. Indeed, this is reflected in a recent survey of members of the Society for Pediatric Radiology, in which a majority of institutions reported always or frequently using high-resolution linear probes for near-field evaluation, but only half of the respondents used far-field evaluation with a linear transducer [8]. As a consequence of the perceived limited penetration capability, the very high-frequency transducers (18-5 MHz), currently used for small parts and lung ultrasound, are even less frequently used for cranial US compared to high-frequency (> 9 MHz) transducers. However, most brain structures are located close enough to the transducer to be explorable in neonates, especially preterm ones. Starting from 2018, in our center, the traditional standard transducers have been supplemented with the use of very high-frequency transducers (18-5 MHz) to perform cranial US in preterm infants. The present case series compares images obtained with these novel applications of the very high-frequency transducers (18-5 MHz) for the neuroimaging of preterm infants ≤ 32 weeks gestation with images obtained from standard medium-frequency (8-5 MHz) micro-convex transducers.

## Methods

We present a case series of US images showing brain abnormalities in preterm infants. The images were obtained simultaneously with both a micro-convex transducer (8-5 MHz) and a very high-frequency (18-5 MHz) transducer. US images are also compared with brain MRI findings at term equivalent age. The machine used was a Philips (Koninklijke Philips N.V., Amsterdam, Netherlands) EPIQ 7 C ultrasound unit with L18-5 MHz linear array transducer and C8-5 MHz micro-convex transducer, whose scanning surface was 6.5 and 2.2 cm in length, respectively. With the very high-frequency transducer, images were acquired by using the “wide screen” function, resulting in larger and trapezoidal images. To improve image quality, preset filters (XRES, SonoCT) were applied, with no post-processing tools nor artificial intelligence–related processing. Spectral Doppler evaluation was excluded because there is no clear evidence to support the routine determination of resistance index or other Doppler parameters in the cerebral arteries to predict brain injury and a long-term neurodevelopmental outcome in the preterm infant [9]. US scans were performed by two technically experienced operators. To minimize inter-operator variability, for each included case, the same operator performed the scans both with the micro-convex transducer and the very high-frequency transducer. To maximize inter-rater reliability, images were examined individually by a third operator, who was blinded to others’ interpretation of neuropathologic findings.

## Results

We included nine neonates who had brain abnormalities visualized on US simultaneously with both a micro-convex probe and a very high-frequency probe. Basic population details are reported in Table S1 (Online Resource). As supplementary information, we included also eight figures from four infants depicting normal cranial US images obtained with the very high-frequency transducer (Online Resource, Fig. S1): note the accurate definition of brain structures. All infants underwent brain MRI at the corrected age (except for one due to death), which confirmed the normalcy of brain structures.

### Intraventricular haemorrhage

**Fig. 1:**
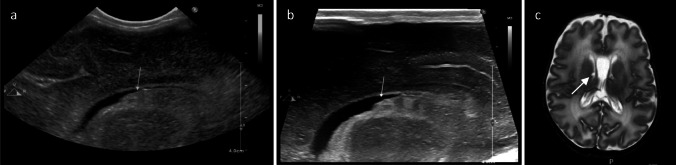
Intraventricular haemorrhage (IVH) grade I in a 29-week-gestation newborn, 10 days of life. **a**, **b** Right parasagittal scans. The definition of the subacute clot (inhomogeneous hyperechoic lesion on the ventricular floor, arrow) is lower using the micro-convex transducer (**a**) compared to the very high-frequency transducer (**b**). MRI performed at term age (**c**): the IVH is no longer visible, while a germinolytic cyst is detected (arrow)

Intraventricular haemorrhage (IVH) in preterm infants usually originates from the immature germinal matrix, from where it can subsequently spread throughout the ventricular system. Its most common grading systems is based on sonographic findings, including the percentage of filling of the volume of the lateral ventricle and the parenchymal involvement [10]. When the haemorrhage remains confined to the subependymal germinal matrix (GMH or grade I IVH), the echodense collection is restricted to the caudothalamic groove. Detecting small GMH on cranial US can be challenging as they cannot be easily differentiated from the hyperechoic choroid plexus. However, the use of very high-frequency transducers enhances the resolution of images and may assist in characterizing cases that remain uncertain when using micro-convex transducers (Fig. 1; Figs. S2 and S3).

**Fig. 2:**
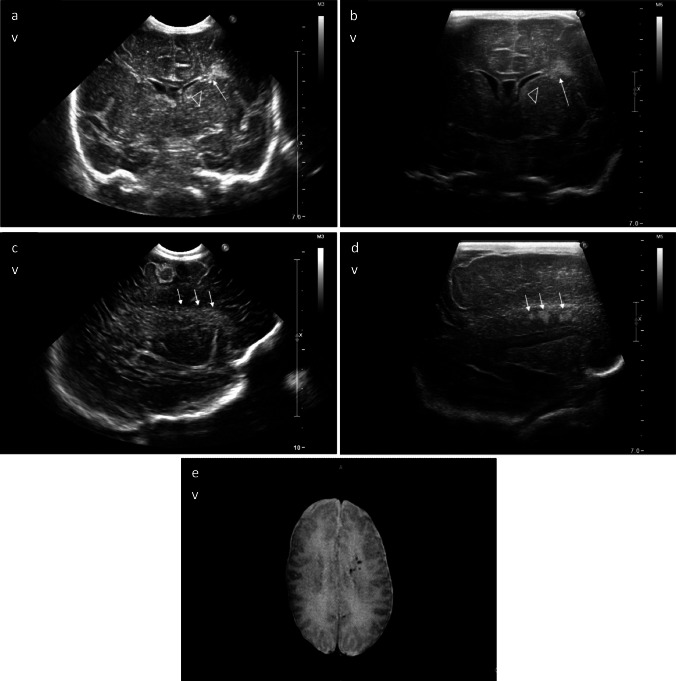
Intraventricular haemorrhage (IVH) complicated by periventricular haemorrhagic infarction (PVHI) in a preterm infant (30-week gestation, 11 days of life). **a**, **b** Coronal scans. The PVHI is presented by an echogenic lesion (white arrow) in the left periventricular white matter, ipsilateral to the IVH (arrowhead). Note that the PVHI is an area of increased echogenicity that is asymmetric, wedge-shaped, and located above the anterior horn of the lateral ventricle. In preterm infants, these lesions should be distinguished from normal periventricular echodensities that are bilateral, symmetric, round-shaped, and located laterally to the anterior horns of the lateral ventricles (as shown in Fig. S1, **a**). **c**, **d** Left parasagittal scans. The micro-convex transducer (**c**) shows subtle hyperechoic lesions in the left periventricular white matter (arrows): however, this scan displays diffuse echogenicity with blurred margins, which can be confused with the typical periventricular hyperechogenic halo of prematurity. By contrast, with the very high-frequency probe (**d**), the PVHI appears inhomogeneous (arrows) with focal hyperechoic areas in contrast with physiological hyperechogenicities (Fig. S1, **g**). **e** MRI at term equivalent age. T2 gradient echo image (axial plane) at the level of the centrum semiovale shows small areas of venous infarctions in the left periventricular white matter, confirming the diagnosis of PVHI.

**Fig. 3:**
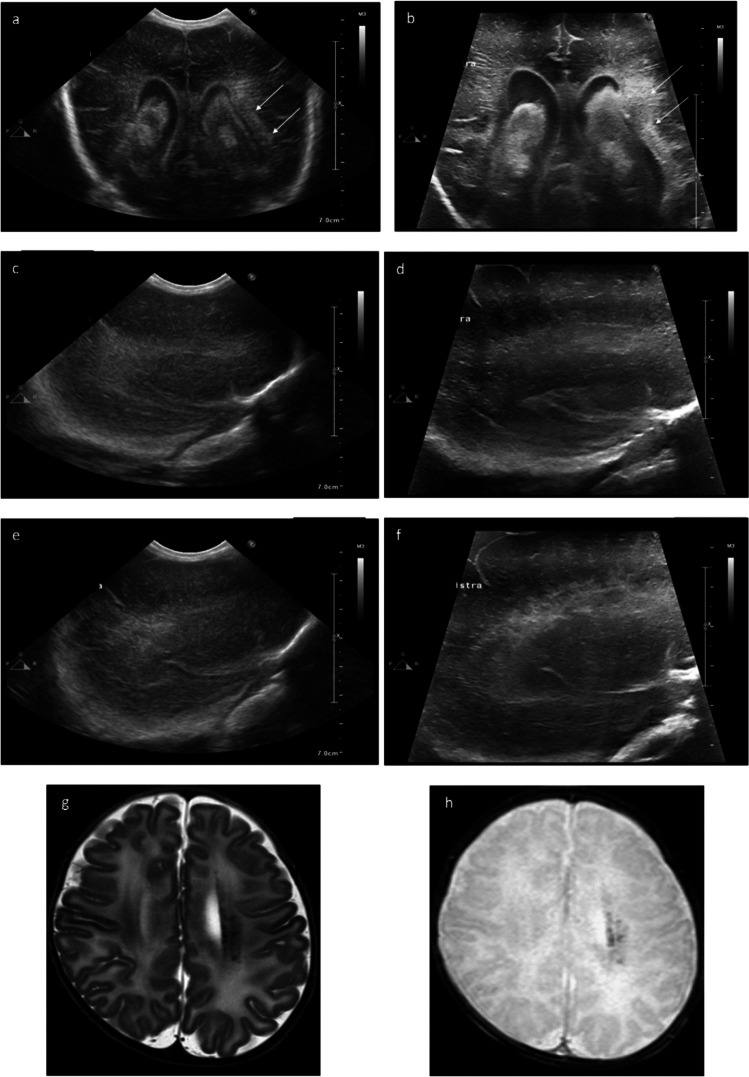
Bilateral intraventricular haemorrhage (IVH) grade 3 with post-haemorrhagic ventricular dilation and left periventricular haemorrhagic infarction (PVHI) in a preterm infant (30-week gestation, 9 days of life). **a**, **b** Coronal scans. Hyperechoic lesions involving the left periventricular white matter appear inhomogeneous (arrows) using the very high-frequency linear transducers (**b**) at a time when cranial ultrasound performed with the micro-convex transducer shows diffuse hyperechogenicities (**a**). **c**, **d** Parasagittal right scans. With the very high-frequency transducer (**d**), the increased echogenicity of the right periventricular white matter is better characterized as homogeneous compared to the micro-convex transducer (**c**). **e**, **f** Parasagittal left scans. The increased echogenicity of the left periventricular white matter (**e**) is similar to the right periventricular white matter (**c**) using the micro-convex probe. By contrast, using the very high-frequency linear transducer, increased echogenicity in the left periventricular white matter appears markedly inhomogeneous, findings compatible with PVHI (**f**). These hyperechoic lesions in the left periventricular white matter were confirmed as PVHI on MRI at term equivalent age (**g**, **h**). **g** Minute lesions with decreased intensity on T2-weighted image (axial plane) at the level of the centrum semiovale; **h** on T2 gradient echo image (axial plane) at the level of the centrum semiovale, localized areas of decreased signal intensity are visible in the left periventricular white matter

While supratentorial IVH is usually easily visible by scanning through the anterior fontanelle, haemorrhages extending into the fourth ventricle, cisterna magna, and subdural spaces are more difficult to detect with this approach. The mastoid view enables better detection of haemorrhages in the posterior fossa (Fig. S4). In addition, scanning through the mastoid fontanelle gives a better overview of the third and fourth ventricle and aqueduct of Sylvius, facilitating accurate diagnosis of post-haemorrhagic hydrocephalus. When the fourth ventricle is dilated because of impairment in cerebrospinal fluid flow, the mastoid fontanelle can help identify the location of the obstruction and distinguishing between obstructive and communicating forms of hydrocephalus. In addition to post-haemorrhagic hydrocephalus, another complication of IVH is periventricular haemorrhagic infarction (PVHI). PVHI is a venous infarction due to the obstruction of the terminal veins consequent to the haemorrhage that impairs blood drainage from the medullary veins. On cranial US, PVHI is characterized by a unilateral or strikingly asymmetric echodensity in the periventricular white matter, ipsilateral to the haemorrhage. The echogenicity of the lesion gradually decreases and changes to echolucency, the ultrasonographic end-stage generally being a poroencephalic cyst or several smaller cystic lesions [11]. In the current study, the very high-frequency transducer allowed an earlier and reliable detection of PVHI compared to micro-convex transducers (Figs. 2 and 3).

### White matter injury

White matter injury (WMI) is the most frequent form of preterm brain injury, affecting up to 50% of very low birth weight infants. Due to improvements in neonatal care, cystic WMI, also referred to as cystic periventricular leukomalacia (PVL), has decreased in incidence, whereas the non-cystic, diffuse form of WMI has become prevalent [7]. The sensitivity of cranial US in the detection of diffuse, non-cystic WMI is poor [10]. However, very high-frequency linear transducers may allow the detection of a wider range of white matter lesions and provide a better evaluation of the nature and extent of white matter abnormalities. Indeed, they can also assist in distinguishing normal white matter echodensities in preterm infants from more inhomogeneous and/or echogenic areas that probably represent WMI. In fact, bilateral symmetrical homogeneous periventricular hyperechogenicities are normal before term equivalent age and are typically seen in early scans [12]. They include blushing around the anterior frontal horn and in the parieto-occipital junction of the lateral ventricles (Fig. S1f), which dissipate without cyst formation or ventricular dilatation. These findings likely reflect maturational processes that typically occur during the final trimester of pregnancy and are attributed to the anisotropic effect of layers of migrating cells along radial glia fibers [13]. The assessment of potential injury in periventricular white matter can be greatly facilitated by imaging through the posterior fontanelle, as it takes advantage of a different insonation angle and can separate anisotropic effect from true increased echogenicity due to pathology [13]. The very high-frequency transducers, providing higher resolution, can also help in distinguishing these maturational changes from WMI, where areas of increased echogenicity are mostly inhomogeneous, present with patchy appearance and bilateral but asymmetric distribution [7]. These lesions may evolve into cystic lesions. Cysts resulting from WMI should not be confused with frontal pseudocysts due to germinolysis which are below the line passing through the upper portions of the anterior horns of the lateral ventricle [14].

**Fig. 4:**
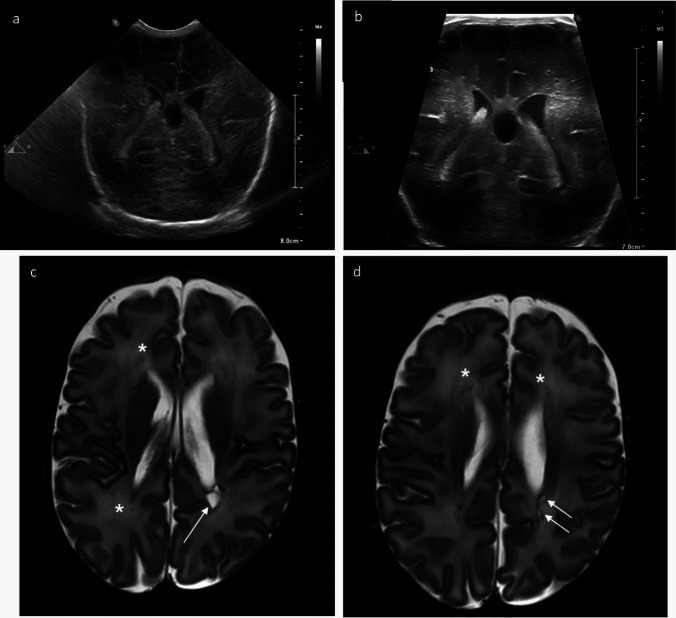
Microcystic periventricular leukomalacia (PVL) and ex vacuo dilatation of lateral ventricles in a preterm infant (29 weeks gestation) with in utero twin-to-twin transfusion. **a**, **b** Posterior coronal scans, 15 days of life. Hyperechoic changes affecting bilateral periventricular white matter that appear diffuse and homogeneous when cranial ultrasound is performed with the micro-convex transducer (**a**). By contrast, using the very high-frequency linear transducer hyperechoic lesions appears more inhomogeneous, and multiple small cysts are visible in the left periventricular white matter (**b**). MRI T2-weighted sequence (**c**, **d**) detected focal punctiform hypointensities in the white matter adjacent to the roof of both middle cells (asterisks). Cavitated lesions in the white matter adjacent to the posterior profile of the left lateral ventricle (arrows). Squared appearance of the posterior aspect of both ventricles and loss of the tapetum, indicative of white matter injury

The duration of periventricular hyperechogenicities correlates with the severity of the injury and with the long-term outcome, even when they do not evolve into cystic lesions. Prolonged hyperechogenicities have been found to predict white matter abnormalities on MRI [7]. Examples of diffuse WMI are shown in Fig. S5. In diffuse, non-cystic WMI, ex vacuo ventriculomegaly may be seen instead of cysts in later scans close to term age (Fig. S5c, d). Figure 4 presents an example of cystic PVL.

### Cerebellum and posterior fossa lesions

**Fig. 5:**
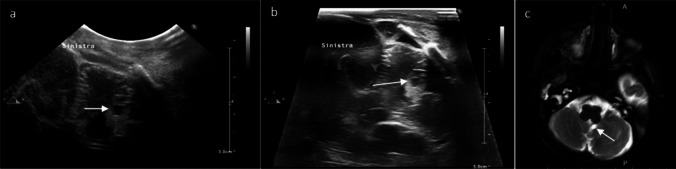
Cerebellar cystic lesion in a moderately preterm infant (gestational age 30 weeks), smaller twin born after dichorionic diamniotic pregnancy complicated by birth weight discordance. **a**, **b** Day 1 of life: coronal ultrasound scan using the left mastoid fontanel as acoustic window. Loss of parenchymal tissue of cerebellar vermis (arrow) is visible with the micro-convex transducer (**a**). Using the very high-frequency transducer (**b**), the echogenicity of the cerebellar parenchyma surrounding the cyst appears abnormally increased, raising suspicion of a prenatal cerebellar lesion with cystic evolution. MRI at term equivalent age (T1-weighted sequence, axial plane at the level of the brainstem) confirmed the cystic lesion (arrow), although its etiology remained undefined

Through the anterior fontanelle, visualization of infratentorial structures is usually suboptimal due to the distance from the transducer to the posterior fossa and to the interposition of the echogenic tentorium. Data on cerebellar infarcts are limited because, without the routine use of mastoid window views, the diagnosis of cerebellar infarctions and sequelae of cerebellar haemorrhages is challenging. However, the distance between the probe and the posterior fossa is smaller in the preterm infant: the use of a very high-frequency transducer allows a sufficient depth penetration to visualize infratentorial structures, in order to exclude haemorrhages and dilation of the aqueduct and the cisterna magna. Furthermore, by utilizing the mastoid fontanelle as an additional acoustic window, as recommended in European guidelines on practices in pediatric neuroradiology [15], the transducer is closer to the posterior fossa structures and approaches them at a different angle, avoiding the tentorium. Also with this approach, very high-frequency transducers can help in the detection of cerebellar lesions (Fig. 5).

Table 1 presents a summary of included brain lesions, comparing US findings using a standard medium-frequency micro-convex transducer, very high-frequency transducers, and magnetic resonance imaging at term corrected age.

**Table 1:** Synthesis of abnormalities detected at neuroimaging: cranial US findings detected with standard medium-frequency micro-convex transducer (second column), very high-frequency linear array transducer (third column), and brain MRI (fourth column)

** Patients (GA at birth) **	**Cranial US—medium-frequency micro-convex transducer**	**Cranial US—very high-frequency linear array transducer**	**Brain MRI**
Figure 1 (29 weeks gestation)	*(Panel a)* Grade I IVH—lower definition of the subacute clot	*(Panel b)* Grade I IVH—better definition of the subacute clot	*(Panel c)* Modest hemosiderin deposition in occipital horns, atria, and posterior sectors of ventricular middle cells was detected. Germinolytic cysts are visible
Figure 2 (30 weeks gestation)	*(Panel a)* The IVH ipsilateral to the PVHI is poorly distinguished *(Panel c)* This scan does not allow a differential diagnosis of PVHI from physiological paraventricular hyperechogenicities in preterm infants	*(Panel b)* The IVH and the PVHI are better defined *(Panel d)* the PVHI appears inhomogeneous with focal hyperechoic areas	*(Panel e)* T2 gradient echo image (axial plane) at the level of the centrum semiovale shows small areas of venous infarctions in the left periventricular white matter, confirming the diagnosis of PVHI
Figure 3 (30 weeks gestation)	*(Panel a)* Homogeneous hyperechoic lesions involving the left periventricular white matter *(Panel c)* The increased echogenicity of the right periventricular white matter is less clear *(Panel e)* Left periventricular white matter is similar to the right periventricular white matter	*(Panel b)* Inhomogeneous hyperechoic lesions involving the left periventricular white matter appear *(Panel d)* The increased echogenicity of the right periventricular white matter is better characterized *(Panel f)* Increased echogenicity in the left periventricular white matter appears inhomogeneous, and multiple small cysts are evident	*(Panel g)* Minute lesions with decreased intensity on T2-weighted image (axial plane) at the level of the centrum semiovale *(Panel h)* T2 gradient echo image (axial plane) at the level of the centrum semiovale: localized areas of decreased signal intensity are visible in the left periventricular white matter
Figure 4 (29 weeks gestation)	*(Panel a)* Hyperechoic changes affecting bilateral periventricular white matter appear diffuse and homogeneous	*(Panel b)* Hyperechoic lesions appear more inhomogeneous and multiple small cysts are visible in the left periventricular white matter, better defining the microcystic PVL	*(Panel c and d)* T2WSE—focal punctiform hypointensities in the white matter adjacent to the roof of both middle cells. Cavitated lesions in the white matter adjacent to the posterior profile of the left lateral ventricle
Figure 5 (30 weeks gestation)	*(Panel a)* Loss of parenchymal tissue of cerebellar vermis is visible	*(Panel b)* Abnormally increased echogenicity of the cerebellar vermis appears around the cystic lesion, suspect of prenatal cerebellar haemorrhage with cystic evolution	*(Panel c)* Cystic lesion at the left inferior paravermian site
Figure S2 (25 weeks gestation)	*(Panel a)* Grade I IVH—extension of the haemorrhage within the lateral ventricle cannot be excluded	*(Panel b)* Grade I IVH—the boundaries of the haemorrhage (arrow) are better depicted. No blood clots are visible within the lateral ventricle	*(Panel c)* Isolated hemosiderin deposition along the ependyma of the left ventricle, result of previous GMH
Figure S3 (27 weeks gestation)	*(Panel c)* Bilateral grade I IVH—haemorrhage is scarcely distinguished from the anterior portion of the choroid plexus	*(Panel d)* Grade I IVH—The boundaries of the hyperechogenic subependymal lesion in the caudothalamic notch are better and can be distinguished from the anterior choroid plexus	*(Panel c)* Normal findings
Figure S4 (27 weeks gestation)	*/*	IVH grade 3 extending to third and fourth ventricle— post haemorrhagic ventricular dilatation: blood residue is visible in third ventricle	/
Figure S5 (24 weeks gestation)	*(Panel a)* Increased echogenicity of the periventricular white matter around the bodies of the lateral ventricles *(Panel c)* Boomerang shape of lateral ventricles	*(Panel b)* The hyperechogenicities appear more inhomogeneous and can be distinguished with the physiologic increased echogenicities of optic radiations *(Panel d)* Triangular shape of lateral ventricles: regular size in frontal horn and ventricular enlargement of the body and occipital horn, suggestive of diffuse WMI	*(Panel e)* Brachycephaly with severe microcrania. Thin callous body with short length

## Discussion

To date, MRI is the gold standard neuroimaging modality to identify central nervous system pathology in prematurity, given its superiority to cranial US in detecting more subtle abnormalities. However, MRI is expensive and requires transport and in some instances sedation; serial scanning remains difficult because access to MRI is limited. Cranial US holds the advantage of being a bedside tool, facilitating safe and reliable serial imaging for the continuous evaluation of injuries over time. Hence, it has emerged as the preferred modality for both initial and sequential neuroimaging in preterm infants. In the current case series, we suggest the use of very high-frequency linear transducers, typically employed for small parts examination and lung ultrasound, for neuroimaging in preterm infants ≤ 32 weeks gestation. Particularly, given the higher resolution demonstrated in our images compared to traditional medium-frequency transducers, the very high-frequency linear transducers could serve as a complementary scanning modality. Because of the lower penetrance of very high-frequency transducers, they are useful especially in preterm infants where the explored areas are in close proximity to the transducer. In our experience, for skilled operators in neonatal neurosonography who primarily use the micro-convex probe, no specific training is required. However, as recommended for performing US with a micro-convex probe [16], the correct adjustment of depth and focus is even more important with the very high-frequency probe. The field of view of the scan should be adjusted to include the maximal depth of the cranium to visualize all cranial contents adequately. The focus should initially be positioned in the posterior fossa, but then moved depending on the findings, adapting it to the region of interest.

There are also limitations to the use of very high-frequency transducers for cranial US. First, the limited access to the brain via the anterior fontanelle: the rectangular field of view obscures the lateral aspect of the brain under parietal bones. Even though the length of the transducer face is 6.5 cm and the anterior fontanelle is usually smaller, the quality of the images is still satisfactory [3, 17]: the areas where most of the abnormalities are located, namely the lateral ventricles and the brain substance in their immediate vicinity, are usually very well visible. However, an important limitation to report is the reduced exploration of peripheral fields due to the larger size of the probe, especially when the anterior fontanelle is small. In particular, in coronal sections, the supero-outer portion of the frontal and parietal lobes is not visualized (Figs. 2b;  4b; Fig. S1a–d; Fig. S3b).

Another theoretical limitation to this approach is the shallow depth associated with very high-frequency transducers compared to the depth achievable with 5-MHz transducers [3, 17]. However, our images show that the 18-5 MHz transducers provide a sufficient view encompassing the scanning field of the basal ganglia, thalami, and also posterior fossa in preterm infants ≤ 32 weeks gestation.

As many US machines now include very high-frequency transducers, performers of cranial US may incorporate this valuable diagnostic tool. While MRI remains the gold standard for neuroimaging in all patients, the utilization of newer transducers operating at very high frequencies may allow for a more precise depiction of findings than previously reported. In conclusion, we recommend considering the use of very high-frequency transducers as a complement to medium-frequency transducers in neonatal cranial US in infants ≤ 32 weeks gestation to enhance the completeness of the diagnostic investigation.

### Supplementary Information

Below is the link to the electronic supplementary material.

Supplementary file1 (DOCX 30.7 kb)

Supplementary file2 (DOCX 7.48 kb)

## Data Availability

No datasets were generated or analyzed during the current study.

## Abbreviations

GMH: Germinal matrix haemorrhage
IVH: Intraventricular haemorrhage
MRI: Magnetic resonance imaging
PVHI: Periventricular haemorrhagic infarction
US: Ultrasound
WMI: White matter injury
