# Supplementary material for: Cranial ultrasound in preterm infants ≤ 32 weeks gestation—novel insights from the use of very high-frequency (18-5 MHz) transducers: a case series
Source: Eur J Pediatr. 2024 Jun 3;183(8):3589–98. doi: 10.1007/s00431-024-05627-y (PMC11263463; doi:10.1007/s00431-024-05627-y)
Supplement: Supplementary file 1 — Supplementary file1 (DOCX 30.7 kb) [file 431_2024_5627_MOESM1_ESM.docx]

|  | **PRENATAL HISTORY** | | | | **PERINATAL HISTORY** | | | | | | | |
| --- | --- | --- | --- | --- | --- | --- | --- | --- | --- | --- | --- | --- |
| Figure  (panels) | Pre-eclampsia | Gestational diabetes | IUGR | Prenatal  Steroid | Gestational age | Delivery | Sex | Birth weight  (g) | Birth weight  (centile) | Apgar 5’ | CRIB II | SNAPPE II |
| 1 | NO | NO | NO | YES, COMPLETE | 29w+2 | VD | F | 1333 | 74° | 8 | 3 | 14 |
| 2 | NO | NO | NO | NO | 30w | VD | M | 1267 | 22° | 9 | 0 | 25 |
| 3 | NO | NO | NO | NO | 30w+5 | CS | F | 1600 | 80° | 7 | 0 | 32 |
| 4 | NO | NO | NO | YES, COMPLETE | 29w+3 | VD | F | 1262 | 70° | 8 | 0 | 25 |
| 5 | NO | NO | YES | YES, COMPLETE | 30w+1 | CS | M | 560 | 0° | 7 | 10 | 50 |
| S1  (a-c,h) | NO | NO | NO | NO | 27w+4 | CS | F | 1010 | 69° | 8 | 10 | 16 |
| S1 (d) | YES | NO | YES | YES, COMPLETE | 25W+5 | CS | F | 498 | 5° | 8 | 14 | 48 |
| S1 (e) | NO | NO | NO | YES, INCOMPLETE | 25w+5 | CS | F | 727 | 57° | 8 | 14 | 52 |
| S1 (f-g) | YES | NO | YES | YES, COMPLETE | 32w | CS | M | 990 | 2° | 7 | NA | NA |
| S2 | NO | NO | NO | NO | 25w | VD | F | 986 | 99° | 7 | 11 | 43 |
| S3 | YES | NO | YES | YES, COMPLETE | 27w+3 | CS | M | 740 | 21° | 9 | 11 | 53 |
| S4 | NO | NO | NO | NO | 27w | CS | M | 925 | 52° | 5 | 12 | 69 |
| S5 | NO | NO | NO | YES, COMPLETE | 24w+3 | VD | M | 660 | 53° | 6 | 14 | 62 |
|  | **AT TIME OF US** | | | | | | | **OUTCOME** | | | | |
|  | PMA | Postnatal days | Body weight (g) | Concomitant diseases | Past disease | Type of ventilation | Drugs | Outcome (discharge) | Days of hospital stay | BPD^a^ | ROP  ≥ 3 | NMO |
| 1 | 30w+4 | 10 | 1460 | NONE | RDS, JAUNDICE | HFNC | CAFFEINE | SURVIVED | 60 | NO | NO | N |
| 2 | 31w+3 | 11 | 1369 | NONE | RDS | NONE | NO | SURVIVED | 43 | NO | NO | N |
| 3 | 32w | 9 | 1434 | NONE | RDS, OLIGURIA | NONE | NO | SURVIVED | 44 | NO | NO | N |
| 4 | 31w+4 | 15 | 1335 | NONE | RDS, JAUNDICE, PDA | CPAP | CAFFEINE | SURVIVED | 93 | NO | NO | MI^b^ |
| 5 | 30w+2 | 1 | 670 | RDS, PPHN, PDA, HYPOGLYCEMIA | NA | MV | AMPICILLIN, GENTAMICIN,  FLUCONAZOLE,  SURFACTANT,  FENTANEST, CAFFEINE, DOPAMINE, DOBUTAMINE, MILRINONE | SURVIVED | 73 | II | NO | ONGOING |
| S1  (a-c,h) | 27w+5 | 1 | 1077 | RDS | NA | CPAP | AMPICILLIN, GENTAMICIN, CAFFEINE, SURFACTANT,  FENTANEST | SURVIVED | 58 | I | NO | ONGOING |
| S1(d) | 25W+6 | 1 | 488 | RDS, HYPOTENSION, CARDIAC HYPERTROPHY, HYPOGLYCEMIA, JAUNDICE | NA | MV | SURFACTANT, DOPAMINE,  DOBUTAMINE,  CAFFEINE,  FENTANEST | DEATH | 25 | NA | NA | NA |
| S1 (e) | 25w+6 | 1 | 747 | RDS, VSD, JAUNDICE | NA | MV | AMPICILLIN, GENTAMICIN, FLUCONAZOLE, DOPAMINE, DOBUTAMINE, CALCIUM, SURFACTANT | SURVIVED | 72 | NO | YES | N |
| S1 (f-g) | 34w+4 | 18 | NA | NONE | RDS, THROMBOCYTOPENIA | NONE | NO | SURVIVED | 30 | NO | NO | N |
| S2 | 27w+1 | 19 | 990 | EVOLVING BPD, PDA | RDS | BiPAP | FLUCONAZOLE,  CAFFEINE | SURVIVED | 98 | I | NO | N |
| S3 | 27w+5 | 2 | 755 | RDS | HYPOGLYCEMIA, JAUNDICE | BiPAP | FLUCONAZOLE, CAFFEINE | SURVIVED | 60 | NO | YES | N |
| S4 | 29w+3 | 19 | 986 | EVOLVING BPD, SEPSIS, ANEMIA | RDS, HYPOTENSION, HYPOCALCEMIA, ANEMIA, HYPERGLYCEMIA | MV | AMPICILLIN, GENTAMICIN, CAFFEINE, FLUCONAZOLE | SURVIVED | 90 | NO | NO | N |
| S5 | 27w+3 | 20 | 786 | EVOLVING BPD, SEPSIS, ANURIA | RDS, PDA | MV | MEROPENEM, DOPAMINE, FENTANEST | DEATH | 180 | III | YES | NA |

BiPAP, Bilevel Positive Airway Pressure; BPD, Bronchopulmonary Dysplasia; CPAP, Continuous Positive Airway P; CS, Cesarean Section; HFNC, High-Flow Nasal Cannula; IUGR, Intra-Uterine Growth Restriction; MI, Moderate Impairment; MV, Mechanical Ventilation; N, Normal; NA, Not Available or Not Applicable; NMO, neuromotor outcome at 24 months of corrected age; PDA, Patent Ductus Arteriosus; PMA, Postmenstrual Age; PPHN, Persistent Pulmonary Hypertension of the Newborn; RDS, Respiratory Distress Syndrome; VD, Vaginal Delivery; VSD, Ventricular Septal Defect

^a^ BPD classification was defined as suggested in Higgins RD, Jobe AH, Koso-Thomas M, Bancalari E, Viscardi RM, Hartert TV, Ryan RM, Kallapur SG, Steinhorn RH, Konduri GG, Davis SD, Thebaud B, Clyman RI, Collaco JM, Martin CR, Woods JC, Finer NN, Raju TNK. Bronchopulmonary Dysplasia: Executive Summary of a Workshop. J Pediatr. 2018 Jun;197:300-308. doi: 10.1016/j.jpeds.2018.01.043. Epub 2018 Mar 16. PMID: 29551318; PMCID: PMC5970962.

^b^ Monoplegia, Gross Motor Function Classification grade 1

**Table S1 Basic Population Details of Infants Included in the Study**. Prenatal history, perinatal data, information at the time of ultrasound, and outcome of the infants included in the study are presented.
