# Supplementary material for: Cranial ultrasound in preterm infants ≤ 32 weeks gestation—novel insights from the use of very high-frequency (18-5 MHz) transducers: a case series
Source: Eur J Pediatr. 2024 Jun 3;183(8):3589–98. doi: 10.1007/s00431-024-05627-y (PMC11263463; doi:10.1007/s00431-024-05627-y)
Supplement: Supplementary file 2 — Supplementary file2 (DOCX 7.48 kb) [file 431_2024_5627_MOESM2_ESM.docx]

**Online Resources**

**Title**

**Cranial ultrasound in preterm infants: novel insights from the use of very high-frequency (18-5 MHz) linear array transducers**

**Journal: European Journal of Pediatrics**

**Authors**

Francesca Miselli^1,2^, Isotta Guidotti^2^, Marianna Di Martino^3^, Luca Bedetti^2^, Chiara Minotti^2^, Eugenio Spaggiari^2^, Giovanni Malmusi^4^, Licia Lugli^2^, Lucia Corso^3^, Alberto Berardi^2,5^

**Correspondence**

Luca Bedetti, MD PhD

Neonatal Intensive Care Unit, Women's and Children's Health Department, University Hospital of Modena, Via del Pozzo, 41124 Modena, Italy

[bedetti.luca@aou.mo.it](mailto:bedetti.luca@aou.mo.it)

+39 059 422 2522

Figure S1

Figure S2

Figure S3

Figure S4

Figure S5

**Figure Legends**

**Fig. S1**

**Panel a** Normal coronal ultrasound scan at the level of the anterior horns of lateral ventricles, in an extremely preterm infant (gestational age 27 weeks, 1 day of life). The interhemispheric fissure demonstrates incomplete folding, along with limited cortical windings observed in the parietal and temporal regions. Echogenicity in the basal ganglia is normal. Note the subcentimetric bilateral symmetric areas of hyperechogenicity (arrows) in the periventricular white matter, adjacent to the frontal horns of the lateral ventricles, a normal finding in preterm infants.

**Panel b** Normal coronal ultrasound scan at the level of the foramina of Monro, same infant shown in panel a. Echogenicity in thalamus and basal ganglia is normal bilaterally. Sylvian fissures are symmetric, and the frontal horns of lateral ventricles and third ventricle demonstrate normal size and morphology.

**Panel c** Normal coronal view passing through the hippocampus, same infant as shown in panels a-b. In preterm infants, the depth penetration of the very high-frequency probe allows exploration of the content of the posterior fossa (cerebellar hemispheres – arrows - and cisterna magna - arrowhead) from the anterior fontanelle.

**Panel d** Normal posterior coronal ultrasound scan at the level of the atrial ventricles in an extremely preterm infant (gestational age 25 weeks, 1 day of life) showing a very smooth interhemispheric fissure and lack of cortical folding. Echogenicity of the periventricular white matter is normal. Note the linear homogeneous symmetrical hyperechogenicities running parallel to the outer edge of the lateral ventricles (arrows), related to the anisotropic effect of layers of migrating cells along radial glia fibers.

**Panel e** Normal midsagittal ultrasound scan in an extremely preterm infant (gestational age 25 weeks, 1 day of life). Detailed image of the entire corpus callosum (arrows), ventral pons (arrowhead) and vermis (long arrow). Cisterns and ventricles are visible as echo-poor areas: from cranial to caudal, the cavum septi pellucidi (a) lies directly below the corpus callosum; the third ventricle (b) is anterior to the thalamus (c); the fourth ventricle (d) is directly anterior to the fastigium; the cisterna magna (e) is below the vermis. The echogenic choroid plexus (asterisk) is attached to the roof of the third ventricle.

**Panel f** Normal left parasagittal ultrasound scan in a moderately preterm neonate (gestational age 32, 18 days of life). Note the physiological blush around the frontal horn and in the parieto-occipital deep white matter (arrows). The blush areas depend on the anisotropic effect of the vascular plexus and radial fibers coursing from the subependyma to the cortex. The internal capsule, separating the lenticular nucleus (a) from the thalamus (b), is barely visible as a moderately hypoechoic area (long arrow).

**Panel g** Normal left lateral parasagittal scan at the level of the insula in a moderately preterm infant (gestational age 32 weeks, 18 days of life) as shown in panel f. Note the homogeneously increased hyperechoic change in white matter external to the lateral ventricle, a normal finding in preterm infants (arrows).

**Panel h** Normal ultrasound scan using the mastoid fontanelle as acoustic window, same infant as shown in panels a-c (gestational age 27 weeks, 1 day of life), coronal view. Panel a, detailed visualization of the cerebellum (vermis (a) and hemispheres (b)), cavum septi (c), aqueduct (short arrow) and cisterna magna (d). Differences in echogenicity and anatomic details between the two hemispheres, due to differences in distance from the transducer, are limited. The transcerebellar diameter can be measured accurately. The lower part of the temporal lobe and hippocampal circumvolution (long arrows) are clearly defined.

**Fig. S2** Intraventricular haemorrhage (IVH) Grade in a 25 weeks’ gestation newborn, 19 days of life. Panels a-b: Left parasagittal scans. Increased focal echogenity (arrow) in the floor of the lateral ventricle, close to the caudothalamic notch. Panel a, curved array transducer: the extension of the haemorrhage within the lateral ventricle cannot be excluded, because of the increased echogenicity of the choroid plexus (arrowhead). Panel b, very high-frequency transducers: the boundaries of the haemorrhage (arrow) are better depicted and can be reliably distinguished from the choroid plexus (arrowhead). No blood clots are visible within the lateral ventricle. Panel c, MRI in the same neonate at term equivalent age showing isolated hemosiderin deposition in the left ventricle (arrow), confirming the diagnosis.

**Fig. S3** Bilateral intraventricular haemorrhage (IVH) in a 27 weeks’ gestation newborn, 2 days of life. Panels a (curved array transducer) and b (very high-frequency linear transducer), coronal sonograms showing increased focal echogenicity in the caudothalamic groove and along the floor of both lateral ventricles (arrows). Panels c and d: parasagittal scans. With the curved array transducer (panel c), the IVH (arrow) can be less distinguished from the anterior portion of the choroid plexus (arrowhead). The boundaries of the hyperechogenic subependymal lesion in the caudothalamic notch (arrow) are better appreciated in with the very high-frequency transducer, and can be distinguished from the anterior choroid plexus (arrowhead, panel d). MRI at term equivalent age yielded normal findings (images not shown).

**Fig. S4** Intraventricular haemorrhage (IVH) grade 3 extending to 3^rd^ and 4^th^ ventricle in a 27 weeks’ gestation newborn, 19 days of life. Coronal scan of the posterior fossa, performed through the mastoid fontanelle with a high-frequency linear transducer showing post haemorrhagic ventricular dilatation: blood residue is visible in 3^rd^ ventricle (arrow) that connects via aqueduct with a dilated 4^th^ ventricle (arrowhead).

**Fig. S5** White matter injury (WMI) in an extremely preterm infant (gestational age 24 weeks, 20 days of life). Panels a-b: posterior coronal ultrasound scan at the level of the choroid plexuses. Increased echogenicity of the periventricular white matter is visible with the curved array transducer around the bodies of the lateral ventricles (arrows, panel a). However, using very high-frequency transducer (panel b), the hyperechogenicities appear more inhomogeneous (arrows), and can be distinguished from the homogeneous symmetrical hyperechogenicities related to the anisotropic effect of layers of migrating cells along radial glia fibers (Fig. S1 panel d, arrows). At term equivalent age (panels c-d, very high frequency linear transducer), ventricles develop an irregular shape. Mild ex vacuo ventriculomegaly is visible with irregular borders without signs of accumulation of cerebrospinal fluid under pressure (i.e. no ballooning). Panel c: posterior coronal scan, boomerang shape of lateral ventricles. Panel d: right parasagittal scan, triangular shape of lateral ventricles: regular size in frontal horn (arrowhead) and ventricular enlargement of the body and occipital horn (arrows), suggestive of diffuse WMI. The diffuse WMI results in low white matter volume on brain MRI performed at the corrected age (panel e: T1-weighted sequence, brachycephaly with severe microcrania. Note thin callous body short in length, arrow)
